# Predicting Synergism of Cancer Drug Combinations Using NCI-ALMANAC Data

**DOI:** 10.3389/fchem.2019.00509

**Published:** 2019-07-16

**Authors:** Pavel Sidorov, Stefan Naulaerts, Jérémy Ariey-Bonnet, Eddy Pasquier, Pedro J. Ballester

**Affiliations:** ^1^CRCM, INSERM, Cancer Research Center of Marseille, Institut Paoli-Calmettes, Aix-Marseille Univ, CNRS, Marseille, France; ^2^Department of Tumor Immunology, Institut de Duve, Bruxelles, Belgium

**Keywords:** chemoinformatics, drug synergy, machine learning, QSAR (qualitative structure-activity relationships), predictive (QSPR) models

## Abstract

Drug combinations are of great interest for cancer treatment. Unfortunately, the discovery of synergistic combinations by purely experimental means is only feasible on small sets of drugs. *In silico* modeling methods can substantially widen this search by providing tools able to predict which of all possible combinations in a large compound library are synergistic. Here we investigate to which extent drug combination synergy can be predicted by exploiting the largest available dataset to date (NCI-ALMANAC, with over 290,000 synergy determinations). Each cell line is modeled using primarily two machine learning techniques, Random Forest (RF) and Extreme Gradient Boosting (XGBoost), on the datasets provided by NCI-ALMANAC. This large-scale predictive modeling study comprises more than 5,000 pair-wise drug combinations, 60 cell lines, 4 types of models, and 5 types of chemical features. The application of a powerful, yet uncommonly used, RF-specific technique for reliability prediction is also investigated. The evaluation of these models shows that it is possible to predict the synergy of unseen drug combinations with high accuracy (Pearson correlations between 0.43 and 0.86 depending on the considered cell line, with XGBoost providing slightly better predictions than RF). We have also found that restricting to the most reliable synergy predictions results in at least 2-fold error decrease with respect to employing the best learning algorithm without any reliability estimation. Alkylating agents, tyrosine kinase inhibitors and topoisomerase inhibitors are the drugs whose synergy with other partner drugs are better predicted by the models. Despite its leading size, NCI-ALMANAC comprises an extremely small part of all conceivable combinations. Given their accuracy and reliability estimation, the developed models should drastically reduce the number of required *in vitro* tests by predicting *in silico* which of the considered combinations are likely to be synergistic.

## Introduction

Drug combinations are a well-established form of cancer treatment (Bayat Mokhtari et al., 2017). Administering more than one drug can provide many benefits: higher efficacy, lower toxicity, and at least delayed onset of acquired drug resistance (Sugahara et al., 2010; Holohan et al., 2013; Crystal et al., 2014). Serendipitous discovery in the clinic has been a traditional source of effective drug combinations (Zoli et al., 2001; Kurtz et al., 2015). Yet systematic large-scale efforts to identify them have only recently been pursued, with a growing number of preclinical experimental efforts to identify synergistic combinations (Zoli et al., 2001; Budman et al., 2012; Lieu et al., 2013; Kashif et al., 2015; Yu et al., 2015; Kischkel et al., 2017) being reported in literature. The sheer number of available and possible drug-like molecules (Polishchuk et al., 2013) and an exponential number of their combinations, however, make the process of finding new therapeutic combinations by purely experimental means highly inefficient.

An efficient way of discovering molecules with previously unknown activity on a given target is using *in silico* prediction methods. Quantitative Structure-Activity Relationship (QSAR) models establish a mathematical relationship between the chemical structure of a molecule, encoded as a set of structural and/or physico-chemical features (descriptors), and its biological activity on a target. Such methods have been successfully used in a wide variety of pharmacology and drug design projects (Cherkasov et al., 2014), including cancer research (Chen et al., 2007; Mullen et al., 2011; Ali and Aittokallio, 2018). QSAR models are traditionally built using simple linear models (Sabet et al., 2010; Pick et al., 2011; Speck-Planche et al., 2011, 2012) to predict the activity of individual molecules against a molecular target. In the last 15 years, non-linear machine learning methods, such as Neural Network (NN) (González-Díaz et al., 2007), Support Vector Machine (SVM) (Doucet et al., 2007) or Random Forest (RF) (Singh et al., 2015), have also been employed to build QSAR models. More recently, QSAR modeling has also achieved accurate prediction of compound activity on non-molecular targets such as cancer cell lines (Kumar et al., 2014).

To extend QSAR modeling beyond individual molecules, the set of features from each molecule in the combination must be integrated. Various ways exist to encode two or more molecules as a feature vector, e.g., SIRMS descriptors (Kuz'min et al., 2008) for properties of combinations or the CGR approach for chemical reactions (de Luca et al., 2012). Rigorous validation strategies for the resulting models have been developed too (Muratov et al., 2012). The most common representation of a drug pair is, however, the concatenation of features from both molecules (Bulusu et al., 2016). On the other hand, modeling drug combinations requires the quantification of their synergy. Several metrics exist to quantify synergy (Foucquier and Guedj, 2015) (e.g., Bliss independence Bliss, 1939, Loewe additivity Chou and Talalay, 1984, Highest single agent approach Greco et al., 1995 or Chou-Talalay Method Chou, 2010). These are implemented in various commercial and publicly available software kits for the analysis of combination data, e.g., Combenefit (Di Veroli et al., 2016), CompuSyn (http://www.combosyn.com) or CalcuSyn (http://www.biosoft.com/w/calcusyn.htm).

One major roadblock in drug synergy modeling has been the lack of homogeneous data (i.e., datasets generated with the same assay, experimental conditions and synergy quantification). This has been, however, alleviated by the recent availability of large datasets from High-Throughput Screening (HTS) of drug combinations on cancer cell lines. For instance, Merck has released an HTS synergy dataset (O'Neil et al., 2016), covering combinations of 38 drugs and their activity against 39 cancer cell lines (more than 20,000 measured synergies). This dataset has been used to build predictive regression and classification models using multiple machine learning methods (Preuer et al., 2018). AstraZeneca carried out a screening study, spanning 910 drug combinations over 85 cancer cell lines (over 11,000 measured synergy scores), which was subsequently used for a DREAM challenge (Li et al., 2018; Menden et al., 2019). Very recently, the largest publicly available cancer drug combination dataset has been provided by the US National Cancer Institute (NCI). This NCI-ALMANAC (Holbeck et al., 2017) tested over 5,000 combinations of 104 investigational and approved drugs, with synergies measured against 60 cancer cell lines, leading to more than 290,000 synergy scores (ComboScores).

NCI-ALMANAC datasets have recently been modeled to predict the best growth inhibition of a given drug combination—cell line tuple (Xia et al., 2018). However, the question remains of how well ComboScores can be predicted on each NCI-60 cell line, which is important given that ComboScore-based screening has led to the identification of novel synergistic drug combinations *in vivo* (Holbeck et al., 2017). Here we present a large-scale study addressing this question. We build an individual model for each cell line using the popular RF algorithm (Breiman, 2001). We also build a second model per cell line using XGBoost (XGB for short) (Chen and Guestrin, 2016), a recent machine learning method that has helped to win numerous Kaggle competitions (Chen and Guestrin, 2016) as well as to generate highly predictive QSAR models (Sheridan et al., 2016). We validate these models for commonly-encountered prediction scenarios: e.g., unseen drug combination or unseen drug partner. We also introduce and validate reliability estimation techniques to further improve prediction of drug combination synergy. Lastly, we assess the suitability of NCI-ALMANAC datasets for predictive modeling depending on the screening center where they were generated.

## Methods

### Data

**Figure 1:**
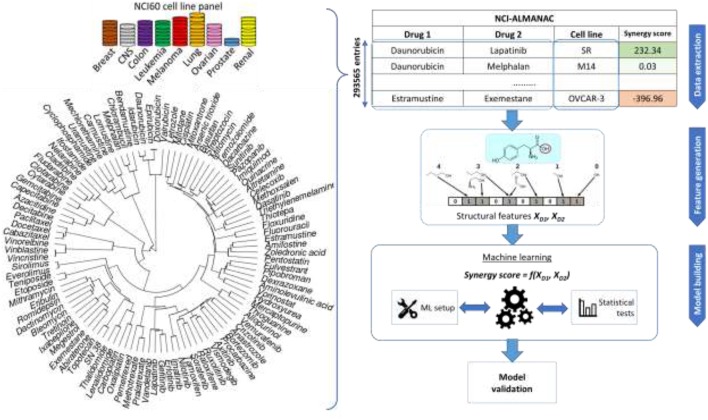
Sketch of the workflow for drug combination modeling. Training data comes from NCI-ALMANAC, which comprises over 290,000 synergy measurements from pairs of 104 drugs tested on the 60 cell lines. Structural and physico-chemical features are calculated for each drug from its chemical structure. Similarity clustering diagram for 104 NCI-ALMANAC drugs is on the left. Each drug is characterized by MFPC features complemented with physico-chemical features, using the Tanimoto score on these features as the similarity metric. Hierarchical agglomerative clustering was carried out (ward.D2 algorithm in the R hclust function). Closely related compounds form tight clusters (e.g., doxorubicin and its analogs, analogs of paclitaxel, etc). By contrast, naturally inorganic compounds such as cisplatin and arsenic trioxide appear as outliers (the highest similarity coefficient to other drugs being 0.156 and 0.125, respectively). The concatenated vectors of the two drugs are the features utilized to build and test predictive models with machine learning techniques. The predictive accuracies of the models are determined by multiple cross-validation experiments.

NCI-ALMANAC is the largest-to-date phenotypic drug combination HTS. It contains the synergy measurements of pairwise combinations of 104 FDA approved drugs on the 60 cancer cell lines forming the NCI-60 panel (Shoemaker, 2006). The drugs include a wide array of small organic compound families, as well as several inorganic molecules (cisplatin and related platinum-organic compounds, arsenic trioxide). A similarity clustering dendrogram (Figure 1) shows the high diversity of the drugs in NCI-ALMANAC. Indeed, only 3 clusters comprising 8 drugs are formed with a Tanimoto score threshold of 0.8 (Vinblastine with Vincristine, Sirolimus and Everolimus, and Daunorubicin-Doxorubicin-Idarubicin-Epirubicin clusters), while the remaining 96 drugs have smaller similarity among them.

NCI-ALMANAC aggregates synergy data from three screening centers: NCI's Frederick National laboratory for Cancer Research (screening center code 1A, 11,259 synergy determinations), SRI International (FF, 146,147 determinations), and University of Pittsburgh (FG, 136,129 determinations). The synergy of drug pairs is measured in these screening centers against the NCI-60 panel, which includes cell lines from nine cancer types: leukemia, melanoma, non-small-cell lung, colon, central nervous system, ovarian, renal, prostate, and breast. In total, synergy is measured for 293,565 drug combination—cell line tuples, which represents a matrix completeness of 91.35%. Each center follows its own protocol, and some drugs are absent from the combination pool depending on the screening center. Since there is no overlap between drug combination—cell line tuples between the three centers, it is not possible to estimate inter-center batch effects, and therefore we must use data from different screening centers separately.

The combination benefit is quantified in NCI-ALMANAC by the so-called ComboScore (a modified version of the Bliss independence model). From the entire dose-response matrix of the considered drug combination and cell line tuple, the gain (or loss) of the effect achieved by the combination over the theoretically expected value if the effect was additive is calculated. Positive values of ComboScore indicate a synergistic effect of the combination, whereas the negative correspond to an antagonistic effect (those purely additive obtain a zero ComboScore).

Further description of NCI-ALMANAC data is available at Supplementary Information.

### Features

For the use in machine learning, the structures of compounds must be encoded as vectors of numerical features known in chemoinformatics as molecular descriptors (Todeschini and Consonni, 2000). Several types of chemical structure features have been considered in this work: (1) Morgan FingerPrints (MFP) are topological descriptors describing the connectivity of the molecular structure, which take values 0 or 1, depending on whether the pattern is present in the molecule or not (Rogers and Hahn, 2010). They have been calculated with RDKit library (Lamdrum, 2015) using the following parameters—length is 256 bits, radius is 2. (2) Morgan FingerPrint Counts (MFPC) are a non-binary version of MFP that takes integer values equal to the number of times the pattern is detected in the molecule (256 features per drug, also calculated with RDKit). (3) MACCS keys encode presence or absence of 166 predetermined substructural fragments as binary vectors (calculated with RDKit). (4) ISIDA fragments encode structure as a vector of numbers of occurrences of substructural fragments of given nature and topology in the molecule (Varnek et al., 2005), which are calculated with ISIDA/Fragmentor (Ruggiu et al., 2015). Only one type of fragments is considered here: sequences of atoms and bonds of length 2 to 6 (1,325 features per drug in total). (5) SIRMS fragments are the number of occurrences of 4-atom fragments of varying topology in a molecule, including bonded and non-bonded atoms (Kuz'min et al., 2008). Calculated with SiRMS python library (github.com/DrrDom/sirms), it led to 1,454 features per drug. In addition to these sets, 7 physico-chemical features are calculated by RDkit: total polar surface area (TPSA), molecular weight, logP, number of aliphatic and aromatic rings, H-bond donors and acceptors.

### Machine Learning (ML) Workflow

Models are built using two ML algorithms: Random Forest (RF) (Svetnik et al., 2003) and Extreme Gradient Boosting (XGBoost; XGB for short) (Sheridan et al., 2016). The entire modeling workflow is sketched in Figure 1. Further details about how ML models were built are available at the Supplementary Information.

### Predictive Performance Metrics

To evaluate the performance of a model, the following metrics are calculated from observed y_*obs*_ and predicted y_*pred*_ ComboScore values:

Root Mean Squared Error (*RMSE)*

$$\begin{array}{l} RMSE=\sqrt{\frac{\sum_{N}{\left(y_{i,\;obs}-y_{i,\;pred}\right)}^{2}}{N}} \end{array}$$

Coefficient of determination (R^2^*)* (Leach and Gillet, 2007)

$$\begin{array}{l} \;R^{2}=1-\frac{\sum_{N}{\left(y_{i,\;obs}-y_{i,\;pred}\right)}^{2}}{\sum_{N}{\left(y_{i,\;obs}-\bar{y_{obs}}\right)}^{2}}=1-\frac{RMSE^{2}}{Var\left(y_{obs}\right)};\\ \bar{y_{obs}}=\frac{1}{N}\overset{N}{\underset{i=1}{\sum }}y_{i,obs} \end{array}$$

Pearson's correlation coefficient (*R_p_*)

$$\begin{array}{l} R_{p}=\frac{\sum_{N}\left(y_{i,\;obs}-\bar{y_{\;obs}}\right)\left(y_{i,\;pred}-\bar{y_{\;pred}}\right)}{\sqrt{\sum_{N}{\left(y_{i,\;obs}-\bar{y_{\;obs}}\right)}^{2}}\sqrt{\sum_{N}{\left(y_{i,\;pred}-\bar{y_{\;pred}}\right)}^{2}}} \end{array}$$

Spearman's rank-order correlation coefficient (*R_s_*)

$$\begin{array}{l} R_{s}=R_{p}\left(\text{rank }y_{obs},\text{ rank}{\;y}_{pred}\right) \end{array}$$

We use R_p_ between observed and predicted values of ComboScore of a dataset not used to train the model as a primary metric of its accuracy. For proper estimation of the generalization error, these metrics are always calculated here on a test set not used to train or select the model.

## Results

### Exploratory Modeling of NCI-ALMANAC Data

**Figure 2:**
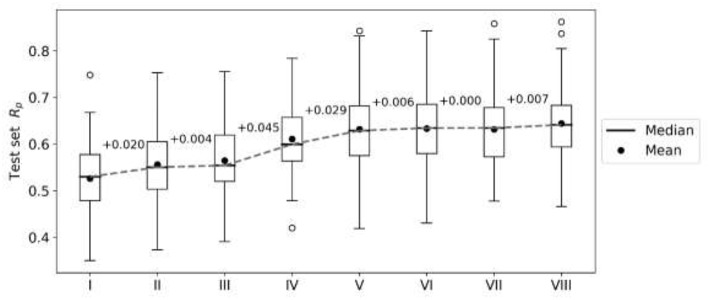
Performance gain across cell lines for each introduced modeling choice during the exploratory analysis of FG data. Each boxplot represents the distribution of the cell line models' test set performances (R_p_) at any given step. Analysis steps are carried out sequentially: I—RF, 1,000 trees with all n features tried to split a node, 80% training set, 20% test set, MACCS (Molecular ACCess System) keys as features; II—MFPC (Morgan fingerprint counts) are used as features instead; III—physico-chemical features are added for each drug; IV—training set rows are duplicated with the reverse order of drugs (data augmentation); V-−90% training set, 10% test set are used instead of the initial 80/20 partition; VI—RF with 250 trees with n/3 features tried to split a node; VII—XGB models with recommended settings; VIII—tuned XGB models. Note that I-V employ RF with same values for its hyperparameters (RF tuned in VI) and V–VIII use the same training and test sets. Modeling choices introducing the largest improvements are the choice of molecular features and the data augmentation strategies.

First, we perform an exploratory modeling on the FG datasets in order to determine optimal settings for synergy prediction by assessing various types of features, data augmentation schemes and machine learning methods. The summary of performance improvements is shown on Figure 2. The best median R_p_ across cell lines for RF was obtained with 250 trees, a third of the features evaluated at each tree node, training data augmentation and MFPC fingerprints complemented by physico-chemical properties (256 and 7 features per drug, respectively). The gain of performance with RF is substantial: the median R_p_ increases from 0.530 (I) to 0.634 (VI).

**Figure 3:**
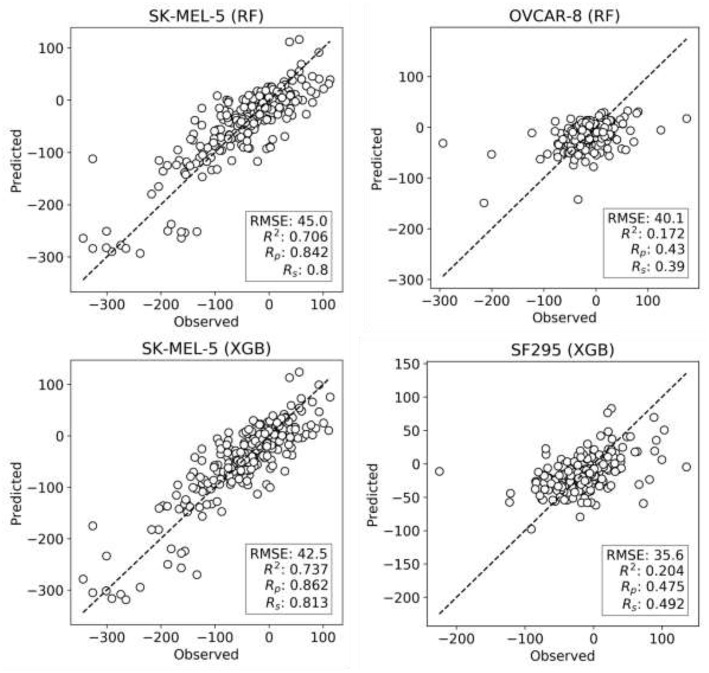
Observed vs. predicted ComboScore for all the drug combinations in the test set. This is presented for the best- and worst-performing models with both ML methods, RF, and XGB (these models correspond hence to the extremes of Figure 2's boxplots VI and VIII, respectively). On the left column, the best-performing cell line models from each method. On the right side, the worst-performing cell line models. All performance metrics are shown. Each point represents a drug combination in that test set.

XGB models are generated with the same features and data set partitions. Changing the machine learning algorithm from RF to XGB does not improve the median test set R_p_, although both minimum and maximum R_p_ are higher with XGB (boxplots VI and VII in Figure 2, respectively). After tuning of XGB hyperparameters per cell line, a small gain in overall performance is obtained: the median R_p_ of tuned XGB rises to 0.641 (boxplot VIII). In comparison, Y-randomization (Tropsha et al., 2003) tests using the same learning algorithm did only obtain a median R_p_ of −0.016 (−0.024 when using RF). Figure 3 shows the degree of accuracy achieved by each algorithm for the best and the worst predicted cell line. The cell lines with the worst predictions (OVCAR-8 for RF and SF295 for XGB) have substantially smaller variance in observed ComboScore than those with the best predictions (SK-MEL-5 for both algorithms).

### Estimating the Reliability of Drug Synergy Predictions

For prospective use of models, it is paramount to calculate not only predicted drug combination synergies, but also how reliable these predictions are (Mathea et al., 2016). With this purpose, we have applied a RF-specific reliability prediction approach, where the degree of agreement between the diverse trees in the forest serves as a reliability score. This is quantified here as the standard deviation (SD) of the RF tree predictions (250 per drug combination and cell line) and referred to as tree_SD. tree_SD has been pointed out as one of the most powerful metrics to assess the reliability of predictions in regression problems (Mathea et al., 2016). We thus assemble test subsets with the 25% most reliable ComboScore predictions per cell line (i.e., combinations with the 25% lowest tree_SD scores). Likewise, we assemble test subsets with 25% least reliable predictions per cell line.

**Figure 4:**
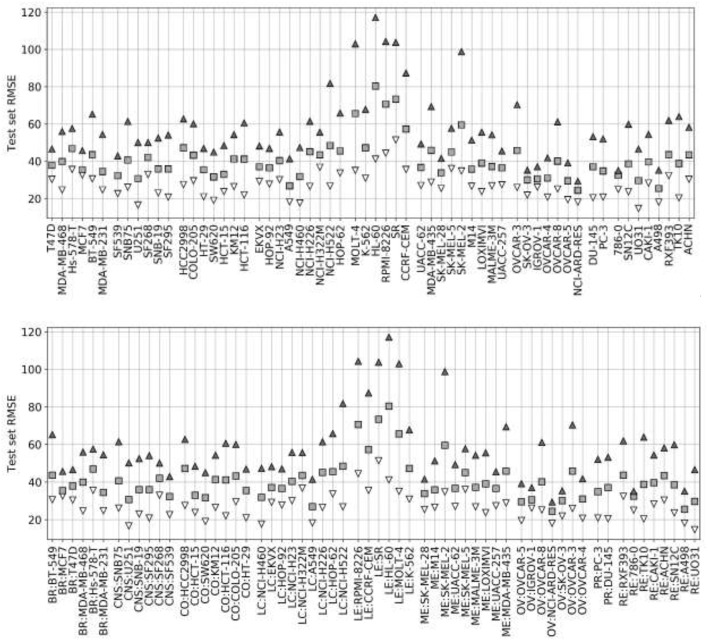
Ten percent test set RMSE of RF cell line models trained on 90% of the FG data. Gray squares represent the model's RMSE on all the test combinations (RF predictions as usual). Black triangles mark the RMSE of the 25% least reliable (highest tree_SD) combos, whereas white inverted triangles correspond to the RMSE of the 25% most reliable (lowest tree_SD). In each cell line, the reliability score tracks test RMSE and hence it can be used to identify *a priori* the most accurate predictions. Each cell line name in the horizontal axis is preceded by its cancer type ID: breast (BR), central nervous system (CNS), colon (CO), non-small-cell lung (LC), leukemia (LE), melanoma (ME), ovarian (OV), prostate (PR), and renal (RE).

Figure 4 presents the test set performances of each cell line model on the three scenarios: 25% most reliable predictions, all predictions regardless of estimated reliability and 25% least reliable predictions. The top and bottom 25% predictions in terms of reliability obtain the lowest and highest RMSE in every cell line, which demonstrates the accuracy and generality of tree_SD as a reliability score for drug synergy predictions. Test set RMSE varies greatly across cell lines, e.g., models built on leukemia cell lines obtain in general higher error. This, however, comes from the higher range of ComboScores observed in these cell lines. Indeed, the larger this range, the higher the range of predicted ComboScores is, which combined tend to make RMSE larger. Similar RMSE is only obtained on the K-562 leukemia cell line, which is consistent with the fact that it has the lowest range among leukemia cell lines and similar to that of other cancer types.

**Figure 5:**
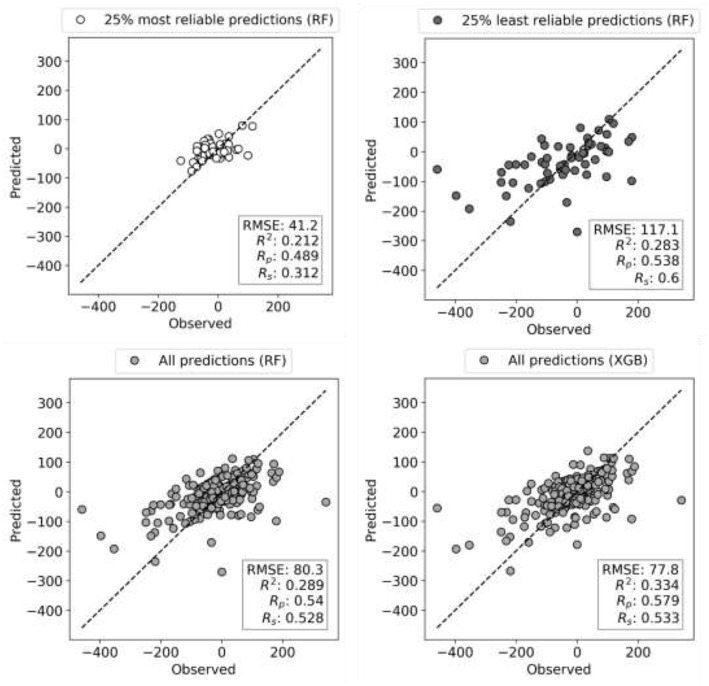
Observed vs. Predicted ComboScore plots for HL-60 leukemia cell line test set (10% of data). Models are built on 90% of FG dataset corresponding to this cell line using RF and XGB methods, both tuned. Each circle is now a drug combination from the entire test set, with its shade of gray indicating one of the three scenarios (as in Figure 4). All performance metrics are displayed in each plot. The subset with the most reliable ComboScore RF predictions (top left plot) achieves half the RMSE of the entire test set (bottom right). Importantly, this is a much larger reduction in RMSE than that introduced by XGB (bottom right) with respect to RF (bottom left). Furthermore, the most reliable predicted ComboScores (top right) obtain a third of the RMSE of the least reliable predictions (top left).

Reliability estimation is evaluated in terms of RMSE rather than R_p_. While RMSE is not as intuitive as correlation, correlations may be misleading when comparing performances of models across test sets with distinct variances. Figure 5 illustrates this issue with the test performances of HL-60 models, which benefit the most from reliability estimation. The test set with the most reliable combinations is predicted with half the RMSE of the entire test set (RMSE of 41 vs. 80) and a third of the least reliable combinations (RMSE of 41 vs. 117). This more accurate prediction can be visually observed too, but the other metrics (R^2^, R_p_, and R_s_) do not capture this increase in accuracy due to substantially different ComboScore variance between the compared test sets. Importantly, RF with reliability prediction provides a much larger reduction in RMSE than that introduced by XGB (bottom right), both with respect to RF without reliability prediction (bottom left). These results strongly suggest that, in cases where it is not necessary to test all positive predictions (here synergistic drug combinations), selecting the most reliable predictions is more effective than using the most suitable ML algorithm.

### Performance in Predicting Synergies With Drugs Not Included in NCI-ALMANAC

The random data splits that we have used so far may overestimate the model's performance in the case of drug combinations. This would be due to the presence of the two drugs in the combination in both training and test sets, albeit with other partners (Muratov et al., 2012). In order to assess to which extent this is the case, we also carry out Leave-One-Drug-Out (LODO) cross-validation experiments for each cell line. In LODO cross-validation, every combination containing the considered left-out drug is placed in its test set, and the model is built on the remaining combinations tested on that cell line. Thus, there are as many folds as drugs in the dataset. In this way, the LODO cross-validation simulates the model's behavior when presented with a new chemical entity outside of the model's scope, as if it was not included in the dataset.

**Figure 6:**
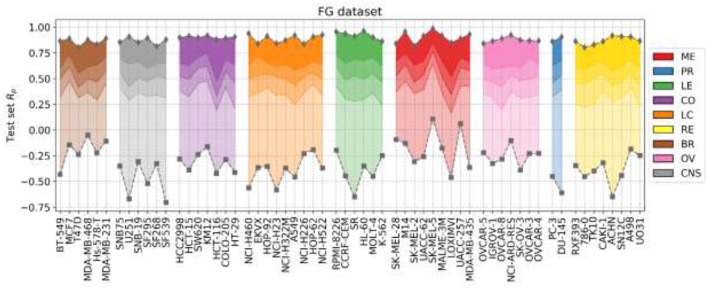
LODO cross-validation results using XGB with the recommended values for their hyperparameters on the FG dataset. Distribution of models' performances is shown by cancer type (color code). Each colored zone represents 25% of models per cell line: from dense zone—top performing 25%; to light zone—bottom quartile. The method performs with at least moderate accuracy (R_p_ > 0.30) in 75% of left-out drugs (the top 3 quartiles) across cell lines. Left-out drugs within the top quartile, darkest shade among the four employed per cell line, are predicted with a R_p_ ranging from 0.471 (HCT-116) to 0.986 (SK-MEL-5). Although there are no large differences in how well different cancer types are predicted, left-out drugs on melanoma (ME, in red) and leukemia (LE, in green) cell lines obtain slightly higher average performance (median R_p_ of drug-out models for corresponding cell lines are 0.554 and 0.524, respectively).

Figure 6 shows the outcome of LODO cross-validation for XGB per cell line. We henceforth use XGB with the recommended values for hyperparameters, as tuning them for each LODO cross-validation fold and cell line is prohibitive and would only provide marginal gains (see Figure 2). LODO results show that combinations associated with 75% of the left-out drugs can be predicted with an accuracy of at least R_p_ = 0.3 against any cell line. This accuracy raises to at least R_p_ = 0.5 for 50% of the left-out drugs. The latter is not much worse than the median R_p_ across cell lines when using 90/10 data partitions (R_p_ = 0.641 as shown in Figure 2's boxplot VIII). k-fold cross-validation results are available for comparison in Supplementary Figure 3.

**Figure 7:**
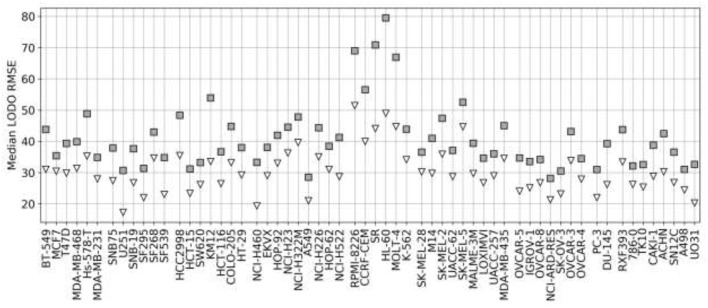
Median RMSE in LODO cross-validation for XGB with the recommended values for their hyperparameters (gray squares) and RF top 25% most reliable predictions (white inverted triangles) for each cell line (grouped by cancer type). As the plot shows, combinations with one left-out drug can be predicted with at least moderate accuracy across cell lines (RMSE < 50 for XGB, RMSE < 40 for RF with reliability estimation; both being approximate thresholds).

Figure 7 shows the analysis for LODO cross-validations in terms of RMSE. About 75% of models demonstrate at least moderate accuracy (RMSE < 50). The exceptions are mostly leukemia cell line models, which obtain higher RMSE due to having the highest variances in ComboScores among cancer types. An important result is that using RF models restricted to the most reliable predictions allows us to reduce the error of prediction further in every cell line (RMSE < 40), in full agreement with the findings from random 90/10 partitions (see Figure 4) and also outperforming the best models without reliability prediction.

Analyzing LODO results per left-out drug instead of per cell line reveals that synergy prediction is much worse for certain left-out drugs in each cancer type. LODO performance of each drug across cell lines is shown in Supplementary Figure 4. This figure shows that models for arsenic trioxide, highly dissimilar to other drugs, have the lowest performance across cell lines and partner drugs (median R_p_ of models concerning this drug is −0.28). Conversely, partners of tyrosine kinase inhibitors, well-represented in these datasets (e.g., Imatinib, Nilotinib or Lapatinib), are predicted with high accuracy (e.g., models for imatinib have median R_p_ = 0.82). Topoisomerase inhibitors (Teniposide and Etoposide) are also among the best-predicted left-out drugs. These *in silico* models could be used to anticipate how the synergies of a drug in combination with its partner drugs would vary across NCI-60 cell lines. However, since high accuracy is only obtained on those left-out drugs well-represented in NCI-ALMANAC, such selectivity predictions should only be accurate for drugs with similar chemical structure to those in NCI-ALMANAC. As models predicting drug-induced cell line response have been shown to improve by integrating drug features with multi-omics cell features (Menden et al., 2013; Xia et al., 2018), we expect that predicting drug synergy across cell lines will also improve by following such multi-task learning approach on this closely related problem.

### Comparing Predictive Models Built With Data From Different Screening Centers

**Figure 8:**
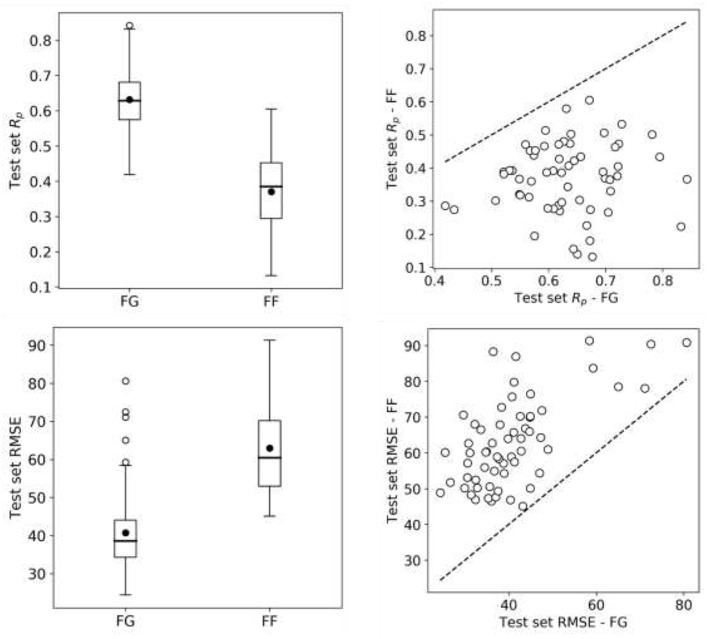
RF model performance comparison for FG and FF datasets. Models are built following the final setup in the exploratory analysis (a 90/10 data partition is employed for each cell line); MFPC with physico-chemical features as well as data augmentation are also used). On the left, boxplots for cell line models test set R_p_'s (top row) and RMSE (bottom row) for both centers data. On the right, R_p_ (top row) and RMSE (bottom row) of models trained on FG dataset against models trained on FF dataset, each point shows the two model performances for the cell line. FF models obtain consistently lower performance than FG models. As the same modeling workflow was used, this strongly suggests that FF data is less predictive than FG data.

So far we have exclusively employed data from the FG screening center, which represents about half of NCI-ALMANAC data. Practically all the remaining ComboScores come from the FF screening center and are also determined with a 3 ×3 grid of non-zero concentrations. Thus, we evaluate here the predictive potential of FF datasets. We start by building RF models from FF data using the same 90/10 partitions as with FG. Surprisingly, FF-based models obtained worse performance in every cell line (Figure 8) and thus were objectively worse at predicting ComboScores.

In trying to understand this unexpected result, we started by investigating whether this was due to modeling differences, but this was not the case. First, FF training sets are slightly larger than FG datasets (see Supplementary Table 1), which theoretically favors better performance on FF. Furthermore, using tuned XGB models led to essentially the same result (median R_p_ of 0.641 for FG vs. 0.368 for FF) as shown in Figure 8 with RF. In addition to these non-linear methods, we also used Elastic Net (EN), but FF models were still substantially less predictive than FG models (median R_p_ of 0.37 for FG vs. 0.23 for FF). When we carried out LODO cross-validations instead of 90/10 partitions, the same trend was observed (Supplementary Figures 5, 6 also show worse performance of FF-based LODO than that of FG-based LODO in Figure 6).

To shed light into this issue, we looked at the only factor that we can compare between these screening centers: the relative growth inhibition (PERCENTGROWTH) induced by a given concentration of a drug tested individually. Interestingly, by counting the different test dates, we observed that FG had on average tested a non-combined drug 3.77 times per cell line, whereas FF almost doubled this number (7.13 times per cell line). A higher number of tests is not in itself worrisome if the growth inhibition of the drug-concentration-cell line tuple is similar between dates. However, if the measurements from these tests are substantially different, this is a problem because the set of ComboScores determined with variable measurements from the same tuple will be inconsistent as well. Consequently, synergy differences between such combinations will not only come from their intrinsic properties, but also from unrelated experimental variability.

**Figure 9:**
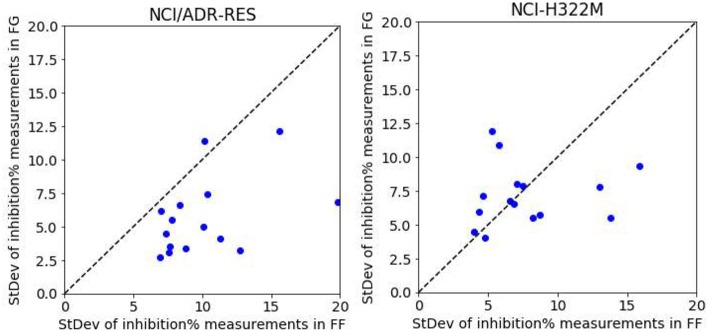
RF model performance comparison for FG and FF datasets. Models are built following the final setup in the exploratory analysis (i.e., a 90/10 data partition is employed for each cell line; MFPC with physico-chemical features as well as data augmentation are also used). We analyzed five drugs (Thioguanine, Chlorambucil, Altretamine, Fluorouracil, and Melphalan) with a high number of different test dates in both centers. On the left, the results with the cell line that is worst predicted by RF with FF data (NCI/ADR-RES with R_p_ = 0.14 in 90/10 partition), which is much better predicted with FG (R_p_ = 0.65, using the same partition). This plot shows the standard deviation of the values of each set from FG against those from FF.

To show that higher growth inhibition variability in FF data results in less predictive models, we analyzed five drugs (Thioguanine, Chlorambucil, Altretamine, Fluorouracil, and Melphalan) with a high number of different test dates in both centers. We first consider the drugs on a cell line were only FG models obtain high average accuracy in predicting synergy (NCI/ADR-RES) and subsequently on another where both FF and FG models are on average predictive (NCI-H322M). On each cell line, each drug has a set of growth inhibition replicates per concentration and screening center (i.e., 15 sets per screening center). The performance on NCI/ADR-RES using FF data is indeed poor (R_p_ = 0.14 in 90/10 partition by RF), but it is much better predicted using FG data (R_p_ = 0.65, using the same partition and method). Fourteen of the fifteen sets have higher standard deviation of growth inhibition with FF data (Figure 9), which is consistent with the lower accuracy in predicting synergy obtained with this dataset. Conversely, we repeated this operation with NCI-H322M where synergy is well-predicted by RF with both FF (R_p_ = 0.61 in 90/10 partition) and FG data (R_p_ = 0.66, on the same partition). The standard deviations from both screening centers are now similar (Figure 9). Taken together, these experiments suggest that the reason why FF data results in less predictive models is the noise introduced in ComboScore determination by larger variability of growth inhibition measurements.

## Discussion

NCI-ALMANAC is an extremely valuable resource for the discovery of novel synergistic drug combinations on NCI-60 cell lines. First, it is by far the largest-to-date HTS of drug combinations, therefore allowing *in silico* models with much higher accuracy and broader domain of applicability in predicting the synergy of other combinations. Second, some of the synergistic drug combinations discovered *in vitro* by NCI-ALMANAC were subsequently tested on human tumor mouse xenografts of the same cell line. 48% of them were also synergistic in at least one of these *in vivo* models (Holbeck et al., 2017), which led to the launch of two clinical trials so far (NCT02211755 and NCT02379416).

In this study, we have found that it is possible to predict the synergy of unseen drug combinations against NCI-60 panel cell lines with high accuracy by exploiting NCI-ALMANAC data. We have established a general ML workflow (types of structural features, data preprocessing strategy, ML method) to generate such models. When trained on FG data, predicted synergies from these models match observable synergies with R_p_ correlations comprised between 0.43 and 0.86 depending on the considered cell line. Incidentally, these regression problems must be highly non-linear, as EN leads to substantially less predictive models than XGB or RF.

Some cell lines and drug combinations can be predicted with higher accuracy than others. For example, models for the SK-MEL-5 cell line perform best with any method (Figure 6). However, if we use RMSE instead of R_p_ to reduce the influence of the ComboScore range, models for the NCI-ARD-RES are now best (gray squares in Figure 7). Another explanatory factor for this variability is the adequacy of the employed ML technique to the problem instance to solve (each cell line constitutes here a different problem instance). Even if training set size, features and classifier are the same, the modeled relationship between drug synergy and features depend on training set composition and cell line properties (implicitly). It is well-established that the performance of supervised learning algorithms varies depending on the problem instance in ways that cannot be anticipated without doing the actual numerical experiments (Fernández-Delgado et al., 2014). LODO cross-validation also revealed both best and worst partner drugs. These differences are mainly due to the number of similar partner drugs. For example, it is difficult to predict synergy of combinations containing arsenic trioxide because its 103 partner drugs are highly dissimilar in terms of chemical structure and physico-chemical properties. Indeed, machine learning from dissimilar data instances tend to be less accurate, although here the dissimilarity can be partial as arsenic trioxide's partner can be similar to other NCI-ALMANAC drugs. On the other hand, combinations containing some other drugs are better represented in NCI-ALMANAC and hence tend to be predicted with higher accuracy. This is the case of various alkylating agents, tyrosine kinase inhibitors and topoisomerase inhibitors (Supplementary Figure 4).

Recent QSAR and drug combination modeling studies have evaluated the application of the latest machine learning algorithms (e.g., XGBoost, Deep Neural Network). These studies have found that these algorithms provide better performance on average across targets than RF. However, these gains are small and hence do not always justify the much greater resources required for hyperparameter tuning (Sheridan et al., 2016; Preuer et al., 2018). Performance gains have also been found small here with NCI-ALMANAC data, as the average test set R_p_ of XGBoost across the 60 cell lines is just +0.007 larger than with RF. An important result is that restricting to the most reliable RF predictions provides much greater predictive accuracy than that introduced by a more suitable learning algorithm (e.g., XGBoost). It is surprising that this powerful technique is so uncommonly used, as has already been pointed out (Sheridan, 2013; Mathea et al., 2016). In fact, we are not aware of any other previous study applying reliability estimation to the prediction of drug synergy on cancer cell lines. Here reliability prediction permitted to reduce the RMSE by up to 50% depending on the cell line. This is particularly exciting for virtual screening problems, where only a small subset of the predictions can be tested *in vitro*. In this scenario, it is useful to identify those combinations that are not only predicted to be synergistic, but also reliable because this should provide higher hit rates. Lastly, highly synergistic combinations predicted with low reliability should also be tested, as the corresponding measurements would be those broadening the applicability domain of future models the most.

We have also found that using FG datasets leads to substantially more predictive models than FF datasets. This result is robust in that it is observed with various types of models (XGB, RF, EN). Moreover, it occurs in spite of the availability of slightly more training data. Further investigation revealed that there are many more measurements of growth inhibition and with greater variability in FF than in FG. This in turn introduces more noise into ComboScore determinations in FF, thus impairing its modeling. Inconsistencies between centers measuring the response of cancer cell lines to drugs have been observed before (Haibe-Kains et al., 2013). There has been intense controversy about the extent, sources and impact of these inconsistencies (Stransky et al., 2015; Geeleher et al., 2016; Safikhani et al., 2016, 2017). In any case, it is clear that data permits the development of predictive models regardless of the screening center (Ammad-ud-din et al., 2014; Covell, 2015; Fang et al., 2015; Naulaerts et al., 2017), as it has also been the case here with NCI-ALMANAC. Owing to this controversy on datasets from multiple screening centers, a better understanding of their limitations and the identification of protocols to generate them with improved consistency has emerged (Haverty et al., 2016). These protocols will ultimately permit that merging datasets from different screening centers result in further predictive accuracy.

## Conclusion

While NCI-ALMANAC measured the synergies of over 5,000 combinations per cell line, this still represents a minuscule part of all conceivable combinations. Even if we restricted ourselves to the set of 12,000 drug molecules estimated to have reached clinical development or undergone significant preclinical profiling (Janes et al., 2018), almost 72 million combinations per cell line would have to be tested *in vitro* to identify the most synergistic among them. Therefore, the developed *in silico* models are of great importance because these can drastically reduce the number of required *in vitro* tests by predicting which of the considered combinations are likely to be synergistic.

## Data Availability

Data available at: https://wiki.nci.nih.gov/display/NCIDTPdata/NCI-ALMANAC.

Code available at: http://ballester.marseille.inserm.fr/NCI-Alm-Predictors.zip.

## Author Contributions

PB conceived the study and wrote the manuscript. PS implemented the software and carried out the numerical experiments with the assistance of SN. All authors commented and proposed improvements to the manuscript.

### Conflict of Interest Statement

The authors declare that the research was conducted in the absence of any commercial or financial relationships that could be construed as a potential conflict of interest.
