# Supplementary material for: Predicting Synergism of Cancer Drug Combinations Using NCI-ALMANAC Data
Source: Front Chem. 2019 Jul 16;7:509. doi: 10.3389/fchem.2019.00509 (PMC6646421; doi:10.3389/fchem.2019.00509)
Supplement: Supplementary file 1 [file Table_1.DOCX]

**Predicting synergism of cancer drug combinations using NCI-ALMANAC data**

Pavel Sidorov, Stefan Naulaerts, Jérémy Ariey-Bonnet, Eddy Pasquier & Pedro J. Ballester

**Supplementary information**

**NCI-ALMANAC composition**

**Supplementary Table 1.** Number of measured ComboScore values in NCI-ALMANAC dataset, grouped by cancer types, cell lines, and screening centers. In total, there are 293,565 ComboScore values across 5050 drug combinations.

| **Cell line** | | | **FG** | | **FF** | **1A** |
| --- | --- | --- | --- | --- | --- | --- |
| **Breast** | BT-549 | 2280 | | 2480 | | 183 |
|  | Hs-578-T | 2256 | | 2471 | | 201 |
|  | T-47D | 2265 | | 2467 | | 185 |
|  | MCF7 | 2289 | | 2518 | | 201 |
|  | MDA-MB-468 | 2274 | | 2475 | | 182 |
|  | MDA-MB-231 | 2288 | | 2513 | | 197 |
| **CNS** | U251 | 2281 | | 2498 | | 199 |
|  | SF295 | 2290 | | 2333 | | 200 |
|  | SNB-19 | 2283 | | 2509 | | 201 |
|  | SNB75 | 2282 | | 2277 | | 201 |
|  | SF268 | 2283 | | 2516 | | 201 |
|  | SF539 | 2274 | | 2414 | | 199 |
| **Colorectal** | SW620 | 2289 | | 2533 | | 201 |
|  | COLO-205 | 2273 | | 2450 | | 199 |
|  | HT-29 | 2279 | | 2495 | | 201 |
|  | HCT-15 | 2290 | | 2510 | | 200 |
|  | KM12 | 2247 | | 2500 | | 199 |
|  | HCT-116 | 2285 | | 2471 | | 192 |
|  | HCC2998 | 2226 | | 2476 | | 196 |
| **Lung cancer** | A549 | 2286 | | 2474 | | 194 |
|  | EKVX | 2287 | | 2505 | | 1 |
|  | HOP-62 | 2285 | | 2355 | | 201 |
|  | NCI-H322M | 2257 | | 2477 | | 199 |
|  | NCI-H226 | 2285 | | 2488 | | 192 |
|  | NCI-H23 | 2283 | | 2510 | | 201 |
|  | NCI-H460 | 2274 | | 2456 | | 201 |
|  | HOP-92 | 2285 | | 2383 | | 137 |
|  | NCI-H522 | 2236 | | 2449 | | 175 |
| **Pros-tate** | PC-3 | 2283 | | 2497 | | 185 |
|  | DU-145 | 2274 | | 2497 | | 198 |

| **Cell line** | | **FG** | **FF** | **1A** |
| --- | --- | --- | --- | --- |
| **Leukemia** | CCRF-CEM | 2247 | 2420 | 190 |
|  | RPMI-8226 | 2129 | 2446 | 200 |
|  | K-562 | 2283 | 2434 | 173 |
|  | SR | 2248 | 2402 | 190 |
|  | MOLT-4 | 2198 | 2415 | 177 |
|  | HL-60 | 2243 | 2234 | 165 |
| **Melanoma** | UACC-257 | 2284 | 2519 | 184 |
|  | LOXIMVI | 2259 | 2318 | 159 |
|  | MDA-MB-435 | 2226 | 2451 | 196 |
|  | UACC-62 | 2281 | 2478 | 199 |
|  | M14 | 2274 | 2461 | 199 |
|  | SK-MEL-2 | 2188 | 915 | 167 |
|  | SK-MEL-5 | 2272 | 2471 | 199 |
|  | SK-MEL-28 | 2283 | 2512 | 201 |
|  | MALME-3M | 2288 | 2499 | 153 |
| **Ovarian** | SK-OV-3 | 2278 | 2513 | 200 |
|  | OVCAR-8 | 2278 | 2489 | 200 |
|  | OVCAR-5 | 2289 | 2515 | 200 |
|  | NCI-ADR-RES | 2289 | 2544 | 169 |
|  | OVCAR-4 | 2256 | 2475 | 193 |
|  | IGROV1 | 2281 | 2479 | 200 |
|  | OVCAR-3 | 2260 | 2399 | 151 |
| **Renal** | SN12C | 2283 | 2491 | 201 |
|  | RXF_393 | 2278 | 2399 | 185 |
|  | A498 | 2289 | 2491 | 200 |
|  | CAKI-1 | 2284 | 2463 | 185 |
|  | TK-10 | 2271 | 2506 | 198 |
|  | ACHN | 2282 | 2527 | 201 |
|  | 786-0 | 2287 | 2462 | 201 |
|  | UO-31 | 2252 | 2452 | 201 |

**NCI-ALMANAC screening centers**

1) NCI Frederick National Laboratory (screening center code 1A) uses the NCI-60 testing protocol (<https://dtp.cancer.gov/discovery_development/nci-60/methodology.htm>), with 5 concentrations per single agent, and 5x3 matrices for combinations. The growth percentage is measured through the classical sulforhodamine cytotoxicity assay (Vichai and Kirtikara, 2006), in which the amount of bound sulforhodamine is observed absorbance measurement at 510 nm wavelength in colorimetry. Therefore, the number of viable cells is proportional to optical density of the dye. 11,259 values in total. Absent drugs: Vemurafenib, Fludarabine.

2) SRI International (FF) uses the modified protocol: drugs are tested in 3 concentrations as single agents, and in a 3x3 concentration matrix for combinations. Cell viability is measured in CellTiter-Glo luminescence assay, luminescence produced is proportional to the number of viable cells. There are 146,177 measured values in total. Absent drugs: Idarubicin, Epirubicin, Eribulin, Abiraterone, Pazopanib, Vismodegib, Crizotinib, Axotinib, Vandetanib, Vemurafenib, Ruxolitinib, Cabazitaxel.

3) University of Pittsburgh (FG) also follows a modified version of the NCI-60 test protocol, with 3x3 concentration matrices for combinations. There are 136,129 measured values from this center. Absent drugs: Doxorubicin, Epirubicin, Idarubicin, Eribulin, Triethylenemelamine.

**NCI-ALMANAC ComboScore**

Expected tumor growth percentage *Z* for cell line *i*, after two-day treatments with drugs *A* and *B* at concentrations *p* and *q*, respectively, is calculated from the observed effect of these drugs as single agents in these concentrations ($Y_{i}^{A_{p}}, Y_{i}^{B_{q}}$, truncated at 100) with the following formula:

$$Z_{i}^{A_{p}B_{q}}=\left\{ \begin{aligned} \min\left( Y_{i}^{A_{p}}, Y_{i}^{B_{q}} \right), if Y_{i}^{A_{p}}\leq0 or Y_{i}^{B_{q}}\leq0 \\ \frac{1}{100}\left( Y_{i}^{A_{p}}\times Y_{i}^{B_{q}} \right), otherwise \end{aligned} \right.$$

The final ComboScore (*CS*) for the cell line and the combination is calculated as the sum of the differences between expected ($Z_{i}^{A_{p}B_{q}}$) and observed ($Y_{i}^{A_{p}B_{q}}$) effects of drug combinations at each concentration:

$${CS}_{i}^{AB}=\sum_{p,q} \left( Z_{i}^{A_{p}B_{q}}-Y_{i}^{A_{p}B_{q}} \right)$$

Since the observed value is the tumor growth percentage, the lower it is in the presence of the drugs, the more synergistic the drugs are. This correspond to more positive ComboScore values.

Supplementary Figure 1 demonstrates the distribution of observed ComboScore values between all three screening centers. University of Pittsburg and SRI International both have a close number of data instances, about 2000 combinations per cell line. NCI’s Frederick National Laboratory, on the other hand, contains less information: about 200 combinations per cell line are tested in this center. The distribution of observed values in first two seems similar, however, SRI International reports more extreme (highly negative or highly positive) ComboScores. In total, 90% of calculated ComboScores are in range between -100 and 100, and only 0.1% are outside of the range from -500 to 500.


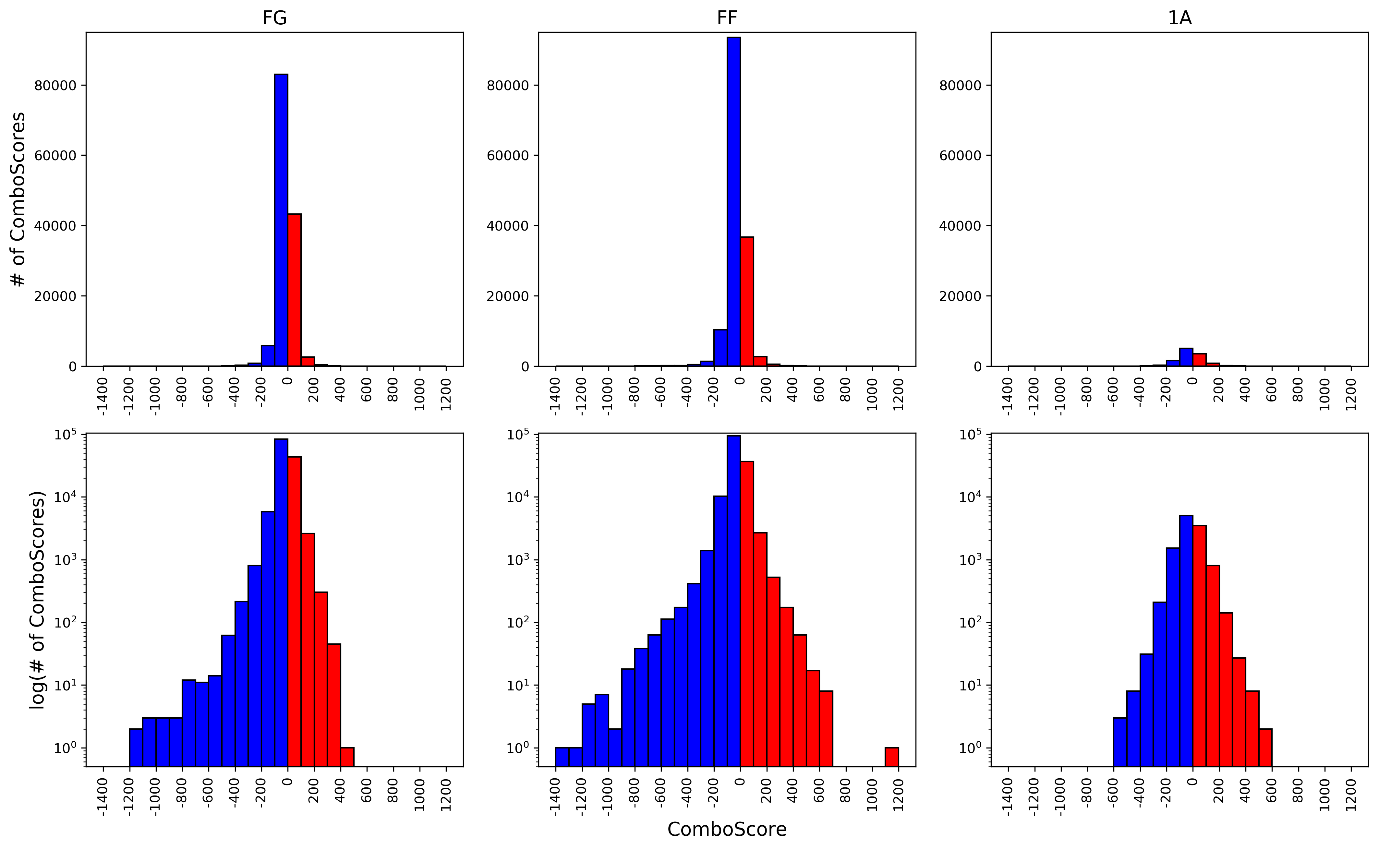


**Supplementary Figure 1.** Histograms of ComboScores measured by each screening center. Number of drug combination – cell line pairs in normal (top row) and logarithmic (bottom row) scales for antagonistic (negative, in blue) and synergistic (positive, in red) effects are presented.


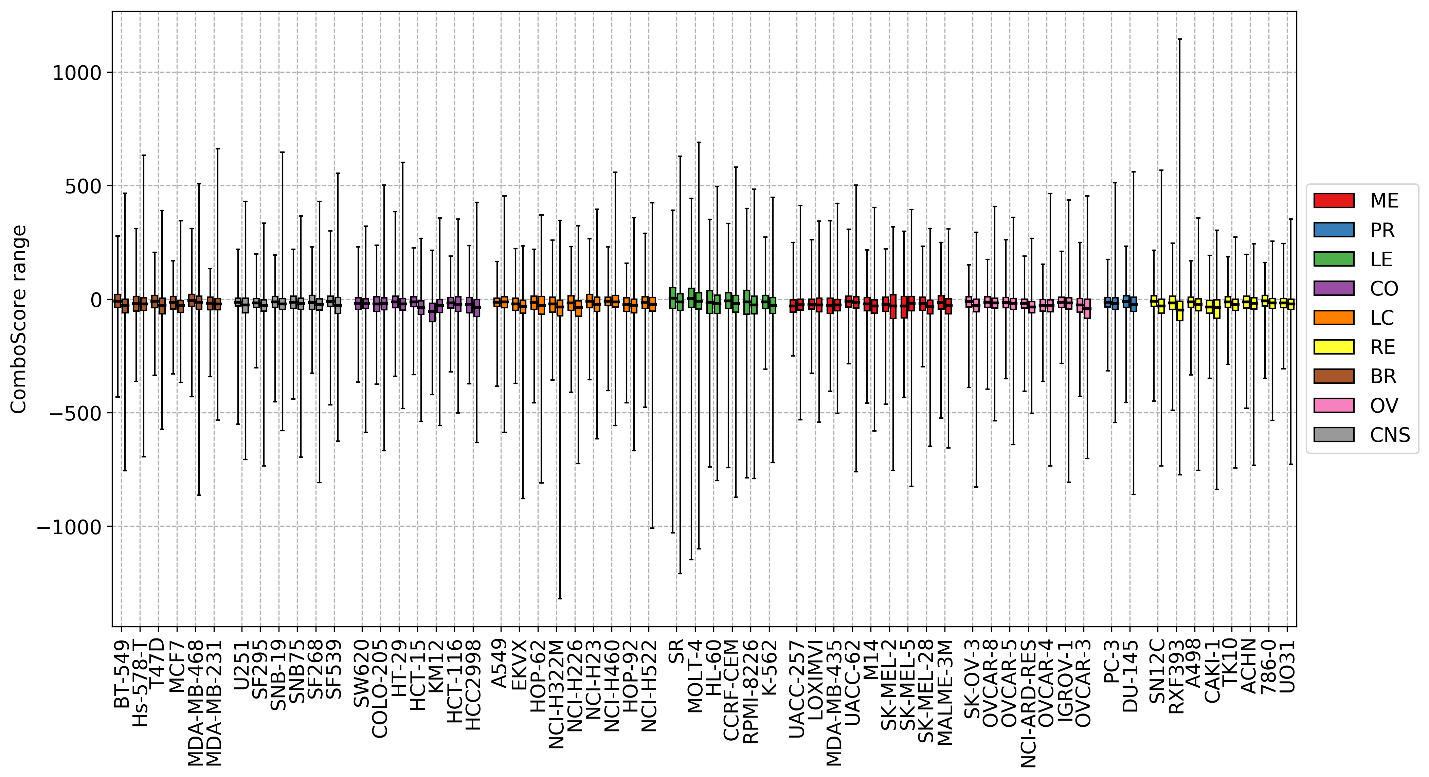


**Supplementary Figure 2.** ComboScore value ranges per cell line. For each cell line, left box corresponds to FG dataset, right box – to FF dataset. The ranges are consistently larger for FF dataset. Boxplots are colored following the cancer type.

**Per-cell line 10-fold cross-validation on FG datasets**

Standard k-fold cross-validation proceeds as following: the dataset is randomly divided in k parts, one is left out as a test set, and other k-1 parts are used to build a model, which is then evaluated on the left-out subset. It is repeated for every subset, so that each instance of the set is predicted exactly once. 10-fold cross-validation has been performed for the RF and XGB cell line models of FG screening center data to confirm the findings of the initial validation on a similar-sized (10% of the set) test sets. All cross-validations of XGB models are carried out with the recommended values for XGBoost’s hyperparameters, as comprehensively tuning in random data partitions only provided marginal gains despite the far higher computing time required.


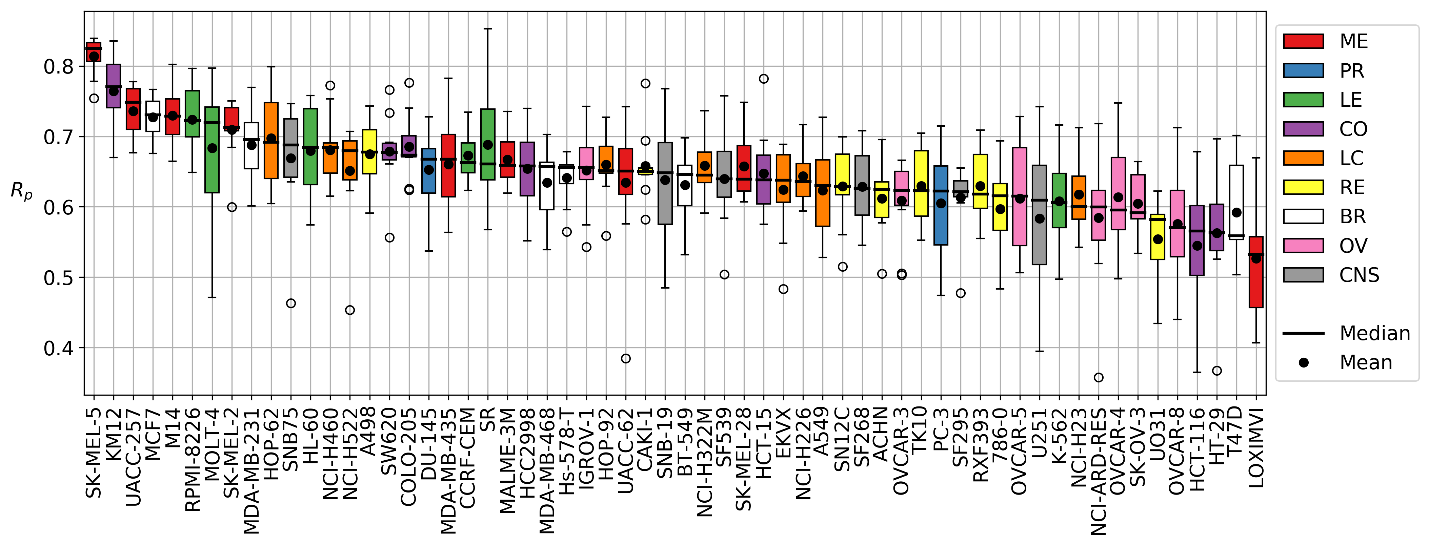


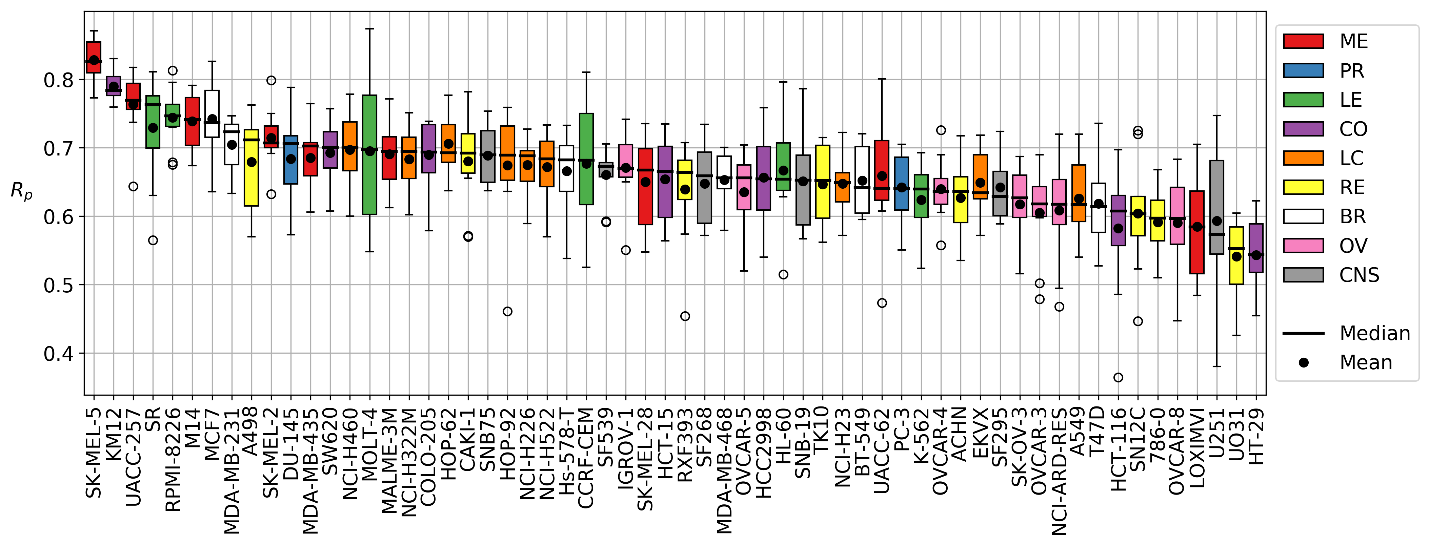


**Supplementary Figure 3.** Test set prediction performance (Pearson correlation R_p_) in 10-fold-cross-validation with Random Forest (top) and XGB (bottom) per cell line. Both algorithms use the recommended values for their hyperparameters. Each boxplot represents the distribution of performances across test folds. Mean and median R_p_ are indicated, boxes are sorted by median performance. Color code indicates cancer tissue type. Some tissue types (such as melanoma ME and leukemia LE) demonstrate higher overall performance than other (e.g. renal cancer RE). Correlation between 10-fold CV results (mean R_p_ between folds) and results of random test set prediction across cell lines (as in the exploratory part) by RF is R_p_=0.56, by XGB R_p_=0.60.

**Per-drug Leave-One-Drug-Out cross-validation on FG datasets**

Leave-one-drug-out cross-validation is carried out in each of the 60 cell lines. Collectively, this results in a RMSE for each left-out drug and cell line pair. Rearranging these results per drug permits assessing how well the left-out drug is predicted across the 60 cell lines (Supplementary Figure 4).


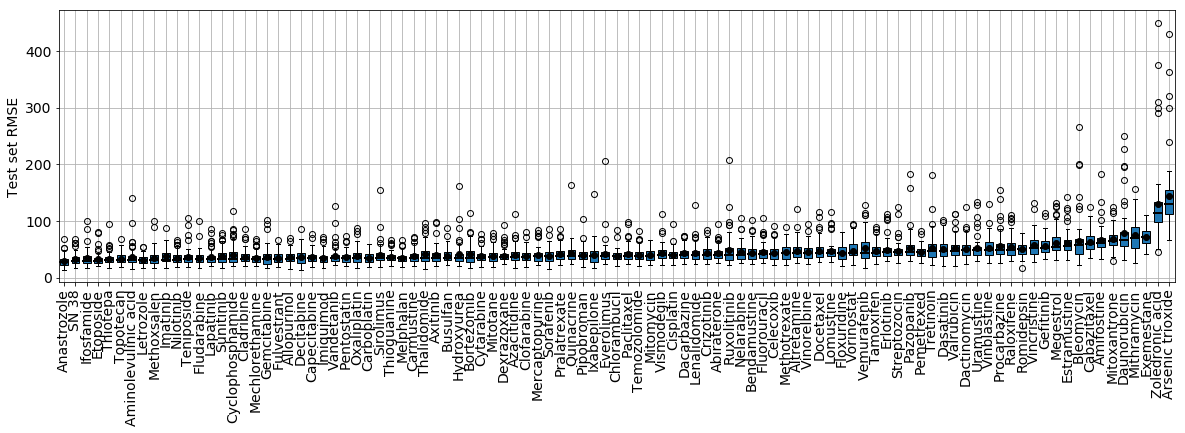


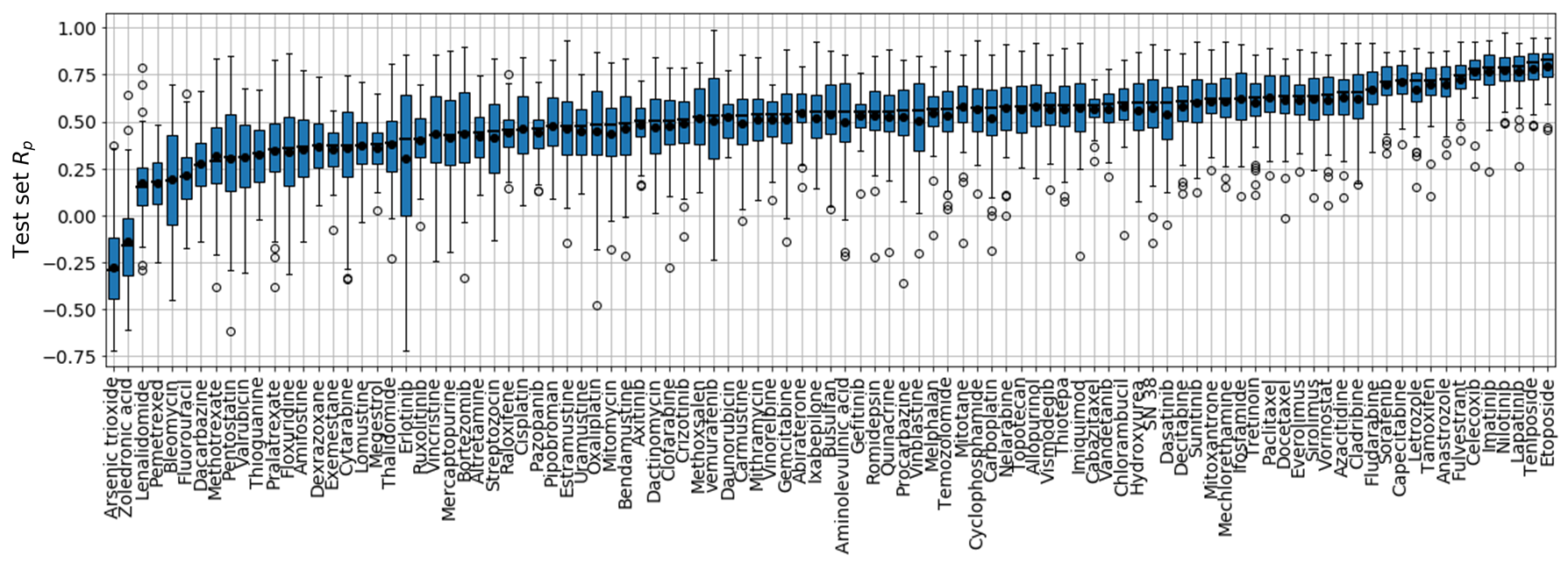


**Supplementary Figure 4**. **(top)** Test set prediction performance (RMSE) in leave-one-drug-out cross-validation with XGB (using the recommended values for their hyperparameters) per left-out drug. Each boxplot represents the distribution of scores across NCI-60 cell lines of all test combinations containing the left-out drug. Mean and median scores are indicated. The boxplots are sorted in order of median RMSE, from lowest to highest. The distribution of predictions is similar to previously discussed RF models: models for Arsenic trioxide and Zoledronic acid have the highest median prediction error (>100), Anastrazole and SN 38 are again in the lead. **(bottom)** Test set prediction performance (R_p_) using the same set of predictions as above. Models for Arsenic trioxide and Zoledronic acid have the lowest median prediction performance (R_p_<0), whereas tyrosine kinase inhibitors (Imatinib, Nilotinib, Lapatinib) and topoisomerase inhibitors (Teniposide, Etoposide) are among the best-predicted left-out drugs.

**Per-cell line Leave-One-Drug-Out cross-validation on FF datasets**

We subjected the FF dataset to the same LODO cross-validation analysis as FG. Supplementary Figure 6 shows the results of LODO cross-validation for FF screening center data. The results of the validation for FF datasets are substantially worse than for FG across all cell lines. Only the top 25% models have average performance R_p_>0.35 (Supp Figure 6). Therefore, we may conclude that there are some inconsistencies within the FF dataset. The most notable occurrence here is the abundance of models obtaining R_p_=1 or -1 across all cell lines. These models correspond to left-out drugs that were only tested with two partners. While this does never occur in the FG dataset, 20 of the 92 drugs tested in the FF are only partnered with doxorubicin and triethylenemelamine (both drugs are absent from FG). Thus, for these drugs the correlation coefficient will be 1 or -1, depending on whether the models are able to correctly learn the synergy trend of these two left-out ComboScores.


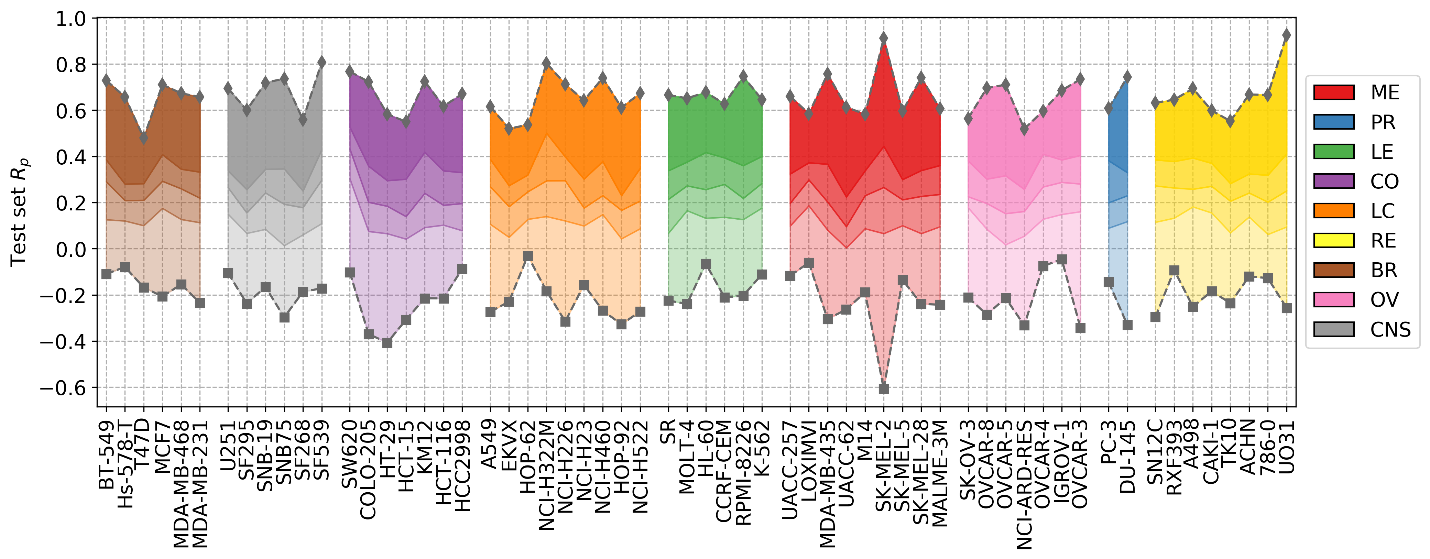


**Supplementary Figure 5.** Leave-one-drug-out cross-validation results with XGB (using the recommended values for their hyperparameters) trained on FF datasets, when using only drugs that have 3 or more partners (i.e. three or more test set instances). Distribution of models’ performances is shown by cancer type (color code). Each colored zone represents 25% of models per cell line: from dense zone – top performing 25%; to light zone – bottom quartile. The performance is not changed compared to the one using all data (Figure 7 in main text), with median R_p_ across cell lines ranging from 0.214 (average for prostate cancer PR, in blue) to 0.254 (average for leukemia LE, in green). Maximum performances range from 0.643 (average for ovarian cancer OV, in pink) to 0.686 (average for brain cancer CNS, in grey) on average across cell lines. Minimum performances range from -0.243 (average for colorectal cancer CO, in purple) to -0.159 (average for breast cancer BR, in brown) on average across cell lines.


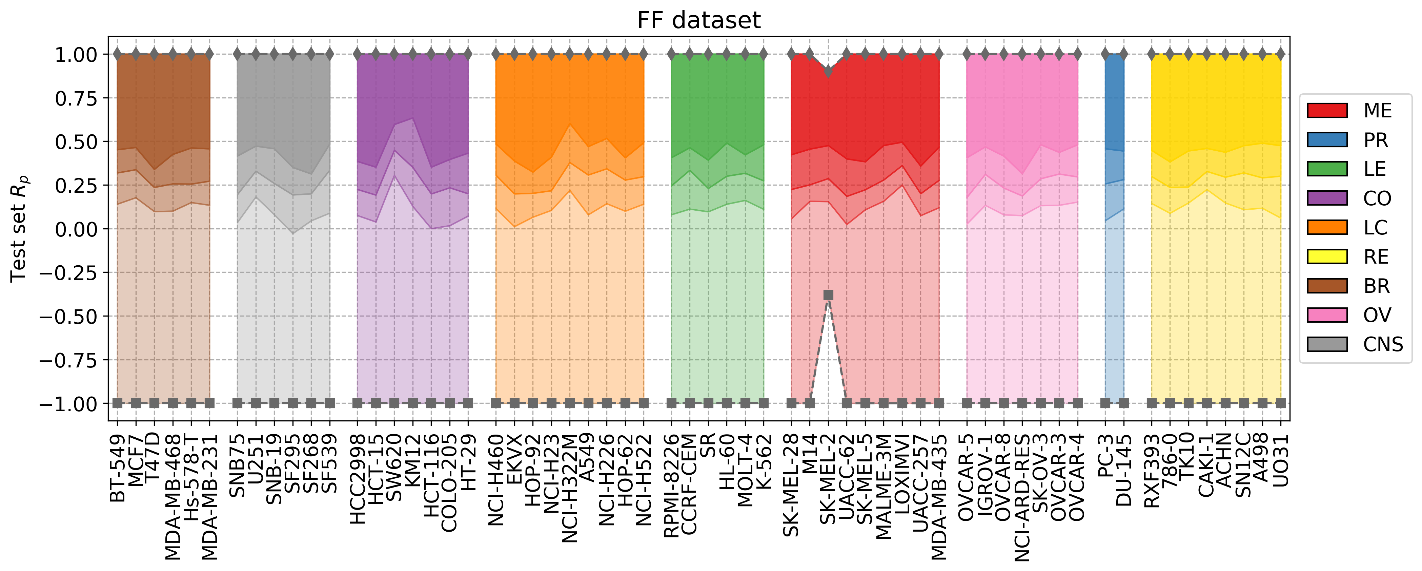


**Supplementary Figure 6.** LODO cross-validation results with XGB (using the recommended values for their hyperparameters) on the FF datasets. Distribution of models’ performances is shown by cancer type (color code). Each colored zone represents 25% of models per cell line: from dense zone – top performing 25%; to light zone – bottom quartile. The method demonstrates lower accuracy on this dataset, with median R_p_ across cell lines ranging from 0.214 (prostate cancer PR, in blue) to 0.254 (leukemia LE, in green), compared to the range from 0.479 (RE) to 0.554 (ME) for FG datasets (see Figure 6). Performances equal to 1 and -1 correspond to drugs for which only two partners are available, thus, only two combinations for that left-out drug are present in the test set of that cell line.


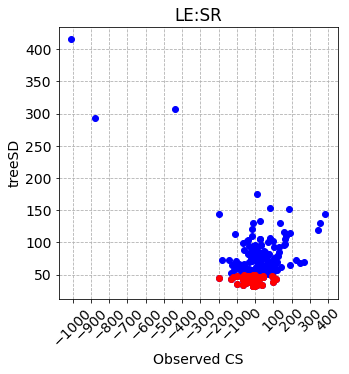

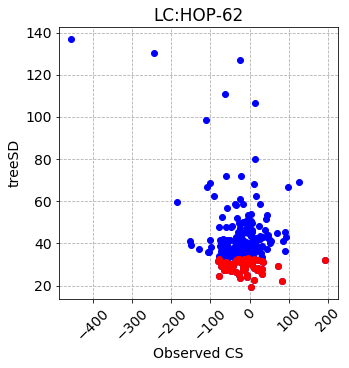

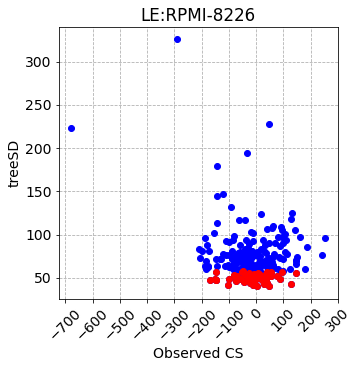


**Supplementary Figure 7.** Examples of cell lines where highly synergistic/antagonistic test set combinations are found within the 25% most reliable predictions (colored in red). The horizontal axis represents observed ComboScore (CS) of test set combinations, y axis their corresponding treeSD values. The 75% of the drug combinations with least reliable CS predictions are colored in blue.

# Methods

## Machine learning workflow

Models are built using two machine learning algorithms: Random Forest (RF) (Svetnik et al., 2003) and Extreme Gradient Boosting (XGBoost; XGB for short) (Sheridan et al., 2016). Both algorithms are here based on regression trees. RF is one of the most widely used methods in QSAR modeling and has several advantages: it is relatively easy to set up, it does not require extensive hyperparameter tuning and its performance is competitive with methods that require such tuning (Sheridan et al., 2016). Here we use the RF implementation from the scikit-learn Python library (Pedregosa et al., 2011). XGB employs the boosting learning paradigm and is often faster to train than RF. In this work, we used the publicly available Python implementation (Chen and Guestrin, 2016) (http://xgboost.readthedocs.io/en/latest/python/). XGB is one of the most popular methods for Kaggle machine learning competitions, and is performing on par with other sophisticated methods such as Deep Neural Network (NN) in QSAR applications, while requiring less setup and hyperparameter tuning as well as being more efficient (Sheridan et al., 2016).

The modeling workflow is sketched in Figure 1. Structural features are generated for each drug, drug pairs are represented by concatenated vectors of features. Five types of features are used: Molecular ACCess System (MACCS) keys (Todeschini and Consonni, 2000), Morgan fingerprints (Rogers and Hahn, 2010), ISIDA fragments (Varnek et al., 2005), SIRMS (Kuz’min et al., 2008), with or without addition of physico-chemical properties (see supplementary information for their description). In theory, the order of the drugs in the concatenated vector should not influence the result. In practice, however, this may introduce a bias into the training set. In order to overcome such bias in drug positioning, a special data augmentation scheme suggested by Preuer et al. (Preuer et al., 2018) is considered: each row of the training set is duplicated by swapping the order of the two feature vectors associated with a drug pair.

Exploratory modeling to optimize the machine learning setup is first carried out on a single random data partition per cell line. The partition is made using scikit-learn library (Pedregosa et al., 2011) function, *train_test_split*. While these partitions are done for each cell line, they are preserved between experiments by fixing the random seed to allow a direct comparison of models’ performance. We also perform experiments with different test set sizes (5%, 10%, or 20% of data), leading to additional data partitions, as well as data preprocessing strategies. For each of these partitions, models are trained on the training set and evaluated on the test set.

The performance of the RF algorithm generally improves marginally with hyperparameter tuning, e.g. (Ballester and Mitchell, 2010), thus we only evaluate five values for the number of trees (100, 250, 500, 750 and 1000 trees) and two values for the number of features considered in splitting a tree node (all and one-third of features). On the other hand, we employ XGB with a recommended set values for its hyperparameters (number of trees 700, maximum tree depth 6, learning rate 0.05, regularization coefficient 0) (Sheridan et al., 2016). Furthermore, we also tune XGB for each cell line model by performing a 5-fold CV on the training set with a grid search of these four hyperparameters: number of trees (200 to 1000), maximum tree depth (5 to 10), learning rate (0.1, 0.05, 0.01) and regularization coefficient (0, 0.0001, 0.001, 0.01). The optimal values are used to build the XGB model with the entire training set of that cell line. To evaluate a model’s performance, the following metrics are calculated from observed and predicted ComboScore values: Root Mean Squared Error (*RMSE)*, Coefficient of determination (*R^2^)*, Pearson’s correlation coefficient (*R_p_*) and Spearman’s rank-order correlation coefficient (*R_s_*), as explained in the paper.

**References**

Ballester, P. J., and Mitchell, J. B. O. (2010). A machine learning approach to predicting protein-ligand binding affinity with applications to molecular docking. *Bioinformatics* 26, 1169–1175. Available at: http://www.ncbi.nlm.nih.gov/pubmed/20236947.

Chen, T., and Guestrin, C. (2016). XGBoost: A Scalable Tree Boosting System. in *Proceedings of the 22nd ACM SIGKDD International Conference on Knowledge Discovery and Data Mining - KDD ’16* (New York, New York, USA: ACM Press), 785–794. doi:10.1145/2939672.2939785.

Kuz’min, V. E., Artemenko, A. G., and Muratov, E. N. (2008). Hierarchical QSAR technology based on the Simplex representation of molecular structure. *J. Comput. Aided. Mol. Des.* 22, 403–421. doi:10.1007/s10822-008-9179-6.

Pedregosa, F., Varoquaux, G., Gramfort, A., Michel, V., Thirion, B., Grisel, O., et al. (2011). Scikit-learn: Machine Learning in Python. *J. Mach. Learn. Res.* 12, 2825–2830.

Preuer, K., Lewis, R. P. I., Hochreiter, S., Bender, A., Bulusu, K. C., and Klambauer, G. (2018). DeepSynergy: Predicting anti-cancer drug synergy with Deep Learning. *Bioinformatics*. doi:10.1093/bioinformatics/btx806.

Rogers, D., and Hahn, M. (2010). Extended-connectivity fingerprints. *J. Chem. Inf. Model.* 50, 742–54. doi:10.1021/ci100050t.

Sheridan, R. P., Wang, W. M., Liaw, A., Ma, J., and Gifford, E. M. (2016). Extreme Gradient Boosting as a Method for Quantitative Structure–Activity Relationships. *J. Chem. Inf. Model.* 56, 2353–2360. doi:10.1021/acs.jcim.6b00591.

Svetnik, V., Liaw, A., Tong, C., Culberson, J. C., Sheridan, R. P., and Feuston, B. P. (2003). Random forest: a classification and regression tool for compound classification and QSAR modeling. *J. Chem. Inf. Comput. Sci.* 43, 1947–58. doi:10.1021/ci034160g.

Todeschini, R., and Consonni, V. (2000). *Handbook of Molecular Descriptors*. Weinheim, Germany: Wiley-VCH Verlag GmbH doi:10.1002/9783527613106.

Varnek, A., Fourches, D., Hoonakker, F., and Solov’ev, V. P. (2005). Substructural fragments: an universal language to encode reactions, molecular and supramolecular structures. *J. Comput. Aided. Mol. Des.* 19, 693–703. doi:10.1007/s10822-005-9008-0.

Vichai, V., and Kirtikara, K. (2006). Sulforhodamine B colorimetric assay for cytotoxicity screening. *Nat. Protoc.* doi:10.1038/nprot.2006.179.
